# Characteristics of soil C:N ratio and δ^13^C in wheat-maize cropping system of the North China Plain and influences of the Yellow River

**DOI:** 10.1038/s41598-017-17060-3

**Published:** 2017-12-04

**Authors:** Huijin Shi, Xiujun Wang, Minggang Xu, Haibo Zhang, Yongming Luo

**Affiliations:** 1 College of Global Change and Earth System Science, Beijing Normal University, Beijing, 100875 China; 2 Ministry of Agriculture Key Laboratory of Crop Nutrition and Fertilization, Institute of Agricultural Resources and Regional Planning, Chinese Academy of Agricultural Sciences, Beijing, 100081 China; 3 Key Laboratory for Coastal Environment Processes and Ecological Remediation, Yantai Institute of Coastal Zone Research, Chinese Academy of Sciences, Yantai, 264003 China; 4 Key Laboratory of Soil Pollution Bioremediation of Zhejiang Province, Zhejiang A&F University, Hangzhou, 311300 China

## Abstract

To better understand the characteristics of soil organic matter (SOM) in the North China Plain, we evaluate the large scale variations of soil organic carbon (SOC), total nitrogen (TN), carbon to nitrogen (C:N) ratio and stable carbon isotopic compositions (δ^13^C) in SOC over 0–100 cm. To assess the influence of the Yellow River, 31 sites are selected from the wheat-maize double cropping system, and grouped into two: 10 sites near and 21 sites far from the river. Our data show that mean soil C:N ratio is low (7.6–9.9) across the region, and not affected by the Yellow River. However, SOC and TN are significantly (P < 0.05) lower in subsoil near the Yellow River (2.0 and 0.2–0.3 g kg^−1^ for SOC and TN) than far away (3.1 and 0.4 g kg^−1^); δ^13^C is significantly more negative below 60 cm near the river (−23.3 to −22.6‰) than far away (−21.8 to −21.4‰). We estimate that the contributions of wheat and maize to SOC are 61.3–68.1% and 31.9–38.8%, respectively. Our analyses indicate that the overall low levels of SOC in the North China Plain may be associated with the low soil C:N ratio and less clay content. The hydrological processes may also partly be responsible, particularly for those near the Yellow River.

## Introduction

Land is an essential nature resource for sustainable agriculture. Maintaining good soil quality is important for high grain production. Soil organic matter (SOM) is a key index of soil fertility, which is closely correlated with crop yields^1,2^. High SOM content often indicates high soil fertility and thus high crop production^3,4^. On the other hand, soil organic carbon (SOC) is a large reservoir for carbon, playing an important role in the global carbon cycle and climate mitigation^5^.

The North China Plain has a long history of intensive cropping, and is an important grain production base in China. This region counts 57% of the nation’s wheat-grow land and provides 66% of the nation’s wheat productivity^6^. The North China Plain and northwest China occupy 44% of the nation’s maize production area and produce 50% of maize grain^7^. Given the fact of relatively larger land with lower soil fertility (thus lower rates of production) in the northwest China, maize yield in the North China Plain would be much above average. Based on these analyses, one may expect that soil fertility thus SOM or SOC in the North China Plain should be above average of China.

However, the data from the Second National Soil Survey (conducted in the 1980s) indicated that the SOC content in topsoil of the Northeastern China (7.9 g C kg^−1^) was lower than the average (11.3 g C kg^−1^)^8^. Our recent studies revealed that mean SOC content was lower than 9.5 g C kg^−1^ in the upper 20 cm of the Hebei Plain and the upper Yellow River Delta^9^. Some other studies carried out in the North China Plain also showed relatively low SOC contents (<9 g C kg^−1^)^10–12^.

Apart from SOC, total nitrogen (TN) is another important index that can indicate soil nutrient condition, with influence on the nitrogen cycle and crop growth^13–15^. Some studies have implied that TN level in the North China Plain is comparable with those in other parts of China^11,16–18^. We postulate that the SOM in the North China Plain may have different characteristics (e.g., the C:N ratio), which can affect soil condition, nutrients cycling and crop growth. For instance, soil C:N ratio has influences on microbial activities thus on SOM decomposition rate^19^, soil health and biogeochemical cycles^13^.

The natural ^13^C abundance (δ^13^C) in SOC is another characteristic of organic matter, which is largely related to sources of organic carbon, and also affected by decomposition^20,21^. Therefore, the isotopic technique has been widely used to study the history of land use and trace the sources of SOC by quantifying relative contributions of C_3_ and C_4_ plants that have different isotopic ^13^C signatures^22–25^. Limited studies have employed this approach to quantify the contributions of wheat and maize to SOC in the northern China^26,27^.

A significant part (i.e., the Yellow River Delta) of the North China Plain is largely influenced by the Yellow River through the hydrological cycle. Our recent study showed that soils near the Yellow River contain higher levels of salts relative to those far away from the river^9,28^. There were evidence that high soil salinity inhibits organic matter decomposition^29,30^, thus one may assume that the SOM level would be greater near the Yellow River. On the other hand, the hydrological cycle that is primarily influenced by the river water and underground water may also have implications for the biogeochemical cycle (e.g., the carbon and nitrogen cycles)^31^. Studies have showed that the Yellow River transports various forms of carbon including dissolved organic carbon (DOC), other chemical materials and biological materials from the land to the Bohai Sea^31,32^. Underground water table is shallow near the Yellow River, implying stronger hydrological impacts near the river^33^.

In this study, we analyze large scale dynamics of SOM properties (i.e., SOC, TN, C:N ratio and δ^13^C) in the upper 100 cm of the North China Plain. To evaluate the influence of the Yellow River, we collect soil samples from the same cropping system (i.e., wheat-maize double cropping), and separate them into two groups: 10 sites near and 21 sites far from the river. The objectives of this study are to better understand the characteristics of SOM in the typical wheat-maize rotation system, and to investigate the impacts of the hydrological processes in association with the Yellow River on soil fertility of the North China Plain.

## Results

### Soil chemical properties

Table 1 shows that mean pH is significantly lower in the sites near the Yellow River than far away (P < 0.05), but average electric conductivity (EC), total dissolved solids (TDS), water-soluble Ca^2+^ and Mg^2+^ contents are significantly higher in the topsoil near the river than far away (P < 0.05, Table 1). For the soil profiles near the Yellow River, there is little vertical variability in soil pH (8.1–8.3), water-soluble Ca^2+^ (93–107 mg kg^−1^) and water-soluble Mg^2+^ (26 to 65 mg kg^−1^). However, mean soil EC and TDS present an increasing trend over depth, i.e, from 271 μs cm^−1^ in 0–20 cm to 445 μs cm^−1^ in 80–100 cm for EC, and from 681 to 1117 mg kg^−1^ for TDS. For the area far away from the Yellow River, our data reveal a small range for pH (8.5–8.7), water-soluble Ca^2+^ (51–72 mg kg^−1^) and Mg^2+^ (16–22 mg kg^−1^) over the 0–100 cm; average EC increases significantly from 141 (above 20 cm) to 299 μs cm^−1^ (below 80 cm), and TDS from 354 to 846 mg kg^−1^ (P < 0.05, Table 1).

**Table 1:** Mean values (standard deviations) of soil pH, electric conductivity (EC), total dissolved solids (TDS), water-soluble Ca^2+^ and Mg^2+^ near the Yellow River and far away from the Yellow River at different soil layer. *Values followed by the same letter (lower case letter within a column or upper case letter between near the Yellow River and far away from the Yellow River) are not significantly different at P < 0.05 based on LSD test. Near means near the Yellow River and far away means far away from the Yellow River.

Depth (cm)	pH		EC (uS cm^−1^)		TDS (mg kg^−1^)		Ca^2+^ (mg kg^−1^)		Mg^2+^ (mg kg^−1^)	
	Near	Far away	Near	Far away	Near	Far away	Near	Far away	Near	Far away
0–20	8.06 (0.18) Ba*	8.48 (0.18) Aa	271 (107) Ab	141 (144) Bb	681 (269) Ab	354 (361) Bc	107 (45) Aa	50 (17) Bb	27 (8) Aa	16 (12) Ba
20–40	8.06 (0.62) Ba	8.67 (0.29) Aa	324 (196) Aab	182 (150) Aab	813 (487) Aab	459 (377) Abc	101(60) Aa	73 (24) Aa	28 (11) Aa	21 (9) Aa
40–60	8.26 (0.24) Ba	8.62 (0.27) Aa	358 (184) Aab	238 (157) Aab	897 (462) Aab	603 (393) Aabc	97 (38) Aa	72 (21) Aa	32 (10) Aa	20 (10) Ba
60–80	8.20 (0.17) Ba	8.57 (0.22) Aa	424 (219) Aab	272 (184) Aab	1062 (547) Aab	763 (424) Aab	97 (34) Aa	62 (14) Bab	33 (18) Aa	20 (10) Ba
80–100	8.24 (0.32) Ba	8.51 (0.25) Aa	445 (227) Aa	299 (196) Aa	1117 (570) Aa	846 (453) Aa	93 (35) Aa	66 (19) Bab	35 (21) Aa	22 (8) Aa

### Spatial variations of SOC and TN

**Figure 1:**
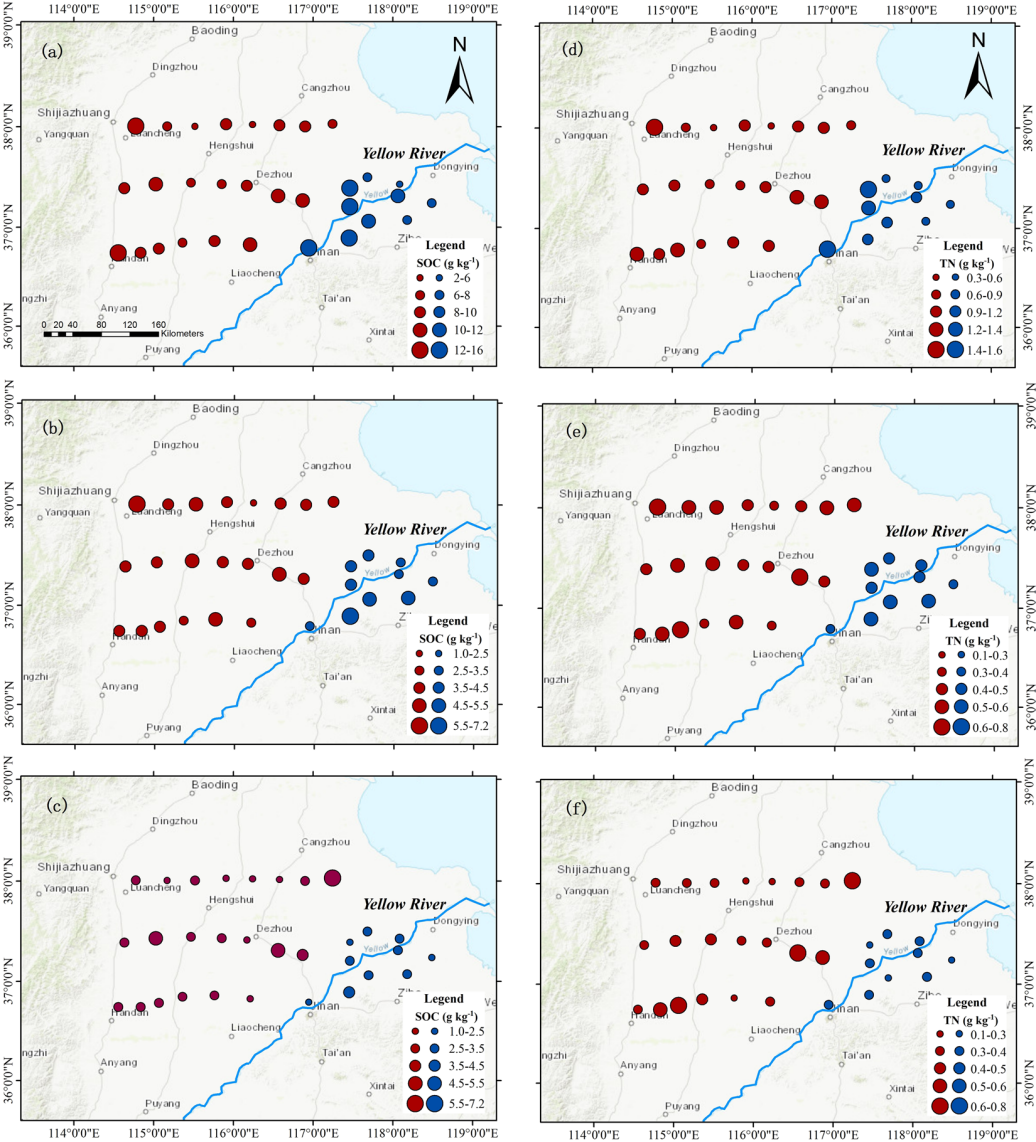
Spatial distributions of the soil organic carbon (SOC) (g kg-1) (left column) and total nitrogen (TN) (g kg-1) (right column) over the 0−20 (**a** and **d**), 20−40 (**b** and **e**) and 40−100 cm (**c** and **f**): near the Yellow River (blue dots) and far away from the Yellow River (red dots). The figure was generated by using ArcMap 10.1 (http://www.esri.com/).

Overall, SOC content in the topsoil (0–20 cm) is larger near the Taihang Mountain and in the upstream of the Yellow River in the study area (Fig. 1a). There is a similar spatial distribution in TN of the topsoil, with the highest values found at the sites in the upstream of the river and near the Mountain (Fig. 1d). However, there is little spatial variation in SOC and TN over the 20–40 cm (Fig. 1b,e). In general, SOC and TN contents are lower in the 40–100 cm near the Yellow River than far away (Fig. 1c,f).

**Figure 2:**
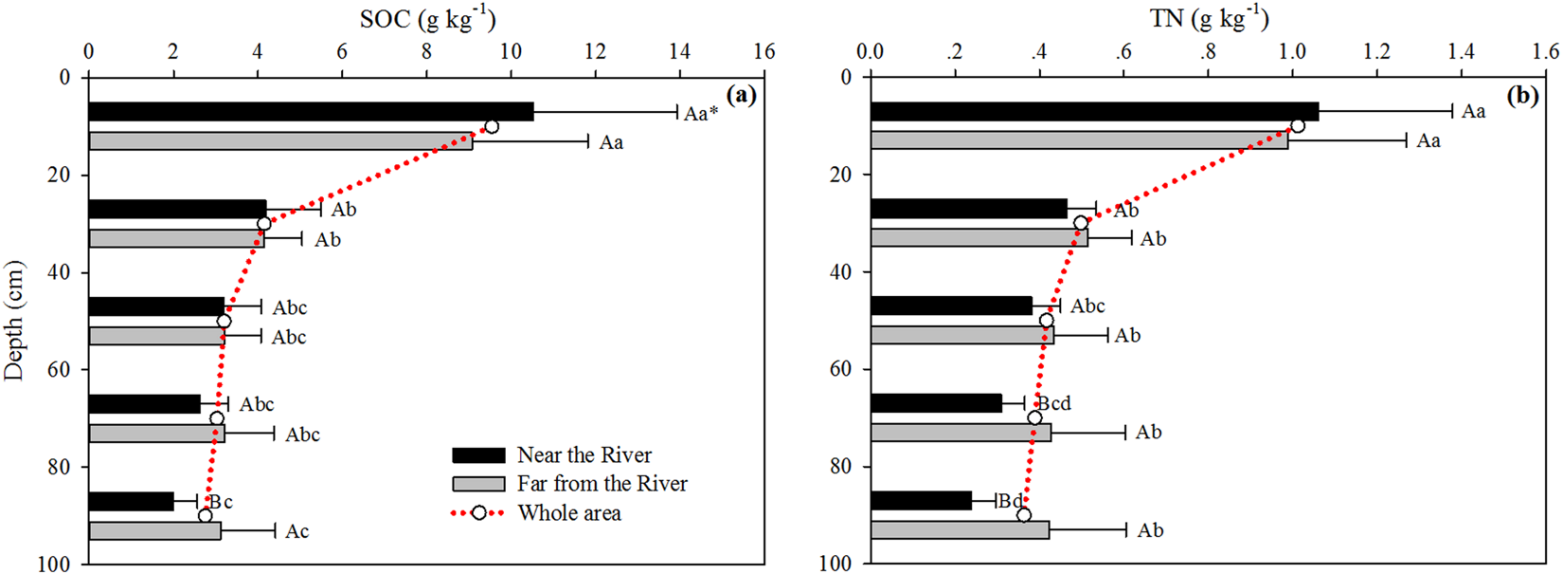
Vertical distributions of mean SOC (**a**) and TN (**b**) in the whole area, near the Yellow River and far away from the Yellow River. *Values followed by the same letter (low case letter between different soil layers or upper case letter between near the Yellow River and far away from the Yellow River) are not significantly different at P < 0.05 based on LSD test.

Figure 2a shows that average SOC below 80 cm is significantly (P < 0.05) lower at the sites close to the river (2.0 g kg^−1^) than far away (3.1 g kg^−1^). Mean TN below 20 cm is lower close to the river (0.24–0.46 g kg^−1^) than far away (0.42–0.48 g kg^−1^), and the difference is significant below 60 cm (P < 0.05, Fig. 2b). As expected, SOC and TN decrease with depth and the differences between 0–20 cm and 20–100 cm is significant (P < 0.05, Fig. 2).

### Spatial variations of C:N ratio and δ^13^C

**Figure 3:**
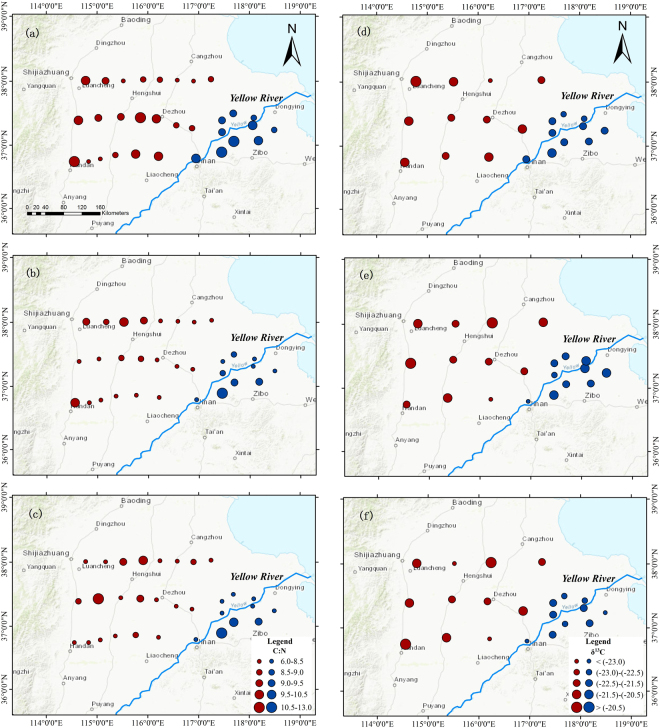
Spatial distributions of C:N ratio (left column) and δC^13^ in SOC (right column) over the 0−20 (**a** and **d**), 20−40 (**b** and **e**) and 40−100 cm (**c** and **f**): near the Yellow River (blue dots) and far away from the Yellow River (red dots). The figure was generated by using ArcMap 10.1.

**Figure 4:**
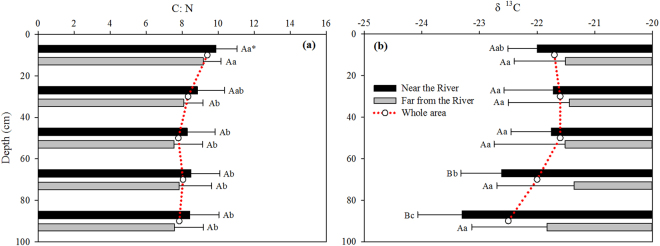
Vertical distributions of mean C:N ratio (**a**) and δC^13^ in SOC (**b**) in the whole area, near the Yellow River and far away from the Yellow River. *Values followed by the same letter (low case letter between different soil layers or upper case letter between near the Yellow River and far away from the Yellow River) are not significantly different at P < 0.05 based on LSD test.

Figure 3 shows the spatial variations of soil C:N ratio and δ^13^C in SOC over the 0–20, 20–40 and 40–100 cm. There is no obvious spatial pattern in soil C:N ratio in the topsoil (Fig. 3a). Relatively high soil C:N ratio in 20–40 cm emerges in northwest and southeast of the study area (Fig. 3b). Soil C:N ratio below 40 cm is generally less than 8.5 (Fig. 3c). Statistical analysis indicates a significant difference in C:N ratio between 0–20 cm and 20–100 cm (P < 0.05), but there is no difference in C:N ratio between near and far from the river (Fig. 4a).

The δ^13^C in SOC over the 0–20 cm is gradually becoming more negative from the foot of the Mountain to the east (near the river) (Fig. 3d). Similarly, ^13^C in the 20–40 cm and 40–100 cm is more depleted at the sites near the River than far away (Fig. 3e,f). A significant difference is found in δ^13^C over the 60–100 cm between near and far away from the river (Fig. 4b, P < 0.05). Interestingly, mean δ^13^C becomes more negative over depth near the river, i.e, from −21.7‰ (0–20 cm) to −22.5‰ (80–100 cm), while δ^13^C far away from the river shows little vertical difference (ranging from −21.4 to −21.8‰).

## Discussion

### Comparison of SOC, TN and soil C:N ratio

Given that both SOC and TN are important parameters for croplands, we compare both with other studies (Table 2). Based on our study and other studies that were conducted in the wheat-maize cropland of the North China Plain, SOC content (<10.5 g kg^−1^) in the topsoil is relatively low, but TN value (0.7–1.6 g kg^−1^) is not so low^11,12,34^. For example, SOC level in the alluvial loess of the North China Plain is lower than in the deposited loess of the Loess Plateau (11.3–11.5 g kg^−1^), but TN is higher relative to those (0.9–1.0 g kg^−1^) in the Loess Plateau^18,34^. Similarly, SOC is lower in the North China Plain relative to Northeast and South China (8.9–25.4 g kg^−1^) with high yields, but TN is comparable (1.0–1.2 g kg^−1^ for Northeast and South China)^16,17^. The decoupling between SOC and TN will not only lead to variations in soil C:N ratio in the cropland, but also has implications for nitrogen release in association with decomposition of SOM.

**Table 2:** Comparison of SOC, TN and C:N for the topsoil in different croplands of China.

Site (experimental or natural)	Region	Annual precipitation (mm)	Annual temperature (°C)	Crop type	FAO soil classification	Sampling year	pH	SOC (g kg^−1^)	TN (g kg^−1^)	C:N	Data sources	Note
The North China Plain (natural cropland)	North China Plain	500–600	12–14	Whiter-wheat and summer-maize	Fluvo-aquic soil	2015	8.3	9.54	1.01	9.45	This study	
Luancheng (experimental site)	North China Plain	536	12.2	Whiter-wheat and summer-maize	Cinnamon soil and Flvuo-aquic soil	2007 2008	—	9–15 9–15	1.2–1.6 1.4–1.8	7.1–7.9 (7.6) 8.3–8.5 (8.4)	Du *et al*. (2010)	Under 4 different treatments
Gaoyi (natural cropland)	North China Plain	513.1	12.6	—	Cinnamon soil and Flvuo-aquic soil	1983 1990 2004	8.35 8.25 8.15	6.13 8.20 10.46	0.70 0.92 1.63	8.72 8.91 6.44	Peng (2011)	Under 7 different soil types
Zhengzhou (experimental site)	North China Plain	632	14.3	Whiter-wheat and summer-maize	Calcaric Cambisol	—	8.3	6.7	0.67	10.0	Zhang *et al*. (2010)	Initial value
Jiangsu-Zhejiang (natural cropland)	Yangze River Delta Plain	1042–1273	14–16	Rice-wheat, rice-rape, wheat-maize, *et al*.	Stagnic Gleyic Cambisol and Gleyic Cambisol	2012–2013	8.1–8.5	7.45	0.73	10.21	Zhang *et al*. (2016)	6 different reclamation duration
Linfen-Yuncheng (natural cropland)	Chinese Loess Plateau	500	12.6	Cereal, vegetables, fruit *et al*.	Haplustepts and Calciustept	2013	—	11.25	1.03	10.82	Yang *et al*. (2016)	
Zhangye (experimental site)	Chinese Loess Plateau	127	7.0	Mono-cropping with maize-wheat-wheat	Anthrosol	—	—	11.5	0.86	13.4	Zhang *et al*. (2010)	Initial value
Urumqi (experimental site)	Northwest China	310	7.7	Mono-cropping with maize-wheat-wheat	Haplic Calcisol	—	8.1	8.8	0.91	10.4	Zhang *et al*. (2010)	Initial value
Songnen Plain (natural cropland)	Northeast China Plain	480–600	—	Maize	Luvic Phaeozems and Chernozem	1980 2005	—	4.1–50.5 (21.4) 4.8–62.6 (25.4)	0.2–2.3 (1.2) 0.3–3.1 (1.2)	4.5–17.7 (10.56) 1.3–18.5 (12.30)	Zhang *et al*. (2011)	
Qiyang (experimental site)	South China	1431	18	Wheat-maize	Eutric Cambisol	1990 1991–1998 1999–2006	5.7	8.85 9.66 11.83	1.07 1.06 1.04	8.27 9.15 11.33	Zhang *et al*. (2009)	Under 8 different treatments

Mean soil C:N ratio over 0–20 cm in our study is 9.4, which is within the range of 6.4–10.0 reported for the cropland of the North China Plain with the same soil type and cropping system (Table 2). Our average C:N ratio is close to those (9.7–9.8) reported for the wheat-maize double cropping in Xuzhou, North China and the mono-cropping with maize-wheat-wheat of Urumqi, Northwest China, but slightly lower than those (10.2–10.8) reported for some other parts close to the North China Plain with a variety of cropping systems, e.g., the Yangze River Delta, East China (rice-wheat, rice-rape, wheat-maize cropping and so on), the Loess Plateau in North and Central China (mono-cropping with maize-wheat-wheat and other cropping systems)^18,34,35^. However, soil C:N ratio is considerably lower in our study than those in the black soil with maize cropping (12.3) in Songnen Plain (Northeast China) and red soil with wheat-maize cropping (11.3) in Qiyang (South China)^16,17^. There is evidence that SOM decomposition rate has a negative relationship with soil C:N ratio^27,36^. Thus, the low soil C:N ratio in the North China Plain may be partly responsible for the low levels of SOM.

### δ^13^C in SOC and contribution of wheat and maize to SOC

The studies on δ^13^C in SOC are limited, most of which focused on the topsoil^20,26,27,37,38^. The δ^13^C in SOC above 20 cm in our study ranges from −23.8 to −20.2‰, which is similar to those (−22.6 to −20.2‰) reported for other wheat-maize croplands of North China^26,27,38^. Overall, δ^13^C shows a little vertical change in the area far from the Yellow River (−21.4‰ to −21.8‰), but becomes more negative with depth near the river. However, limited studies reveal an enriching trend over depth, i.e., in the wheat-maize croplands of Quzhou, Zhengzhou and Yangling^27,38^.

To better understand SOC dynamics in the wheat-maize rotation system, we quantifies the relative contributions of wheat (ƒ_C3_) and maize (ƒ_C4_) to SOC in the North China Plain, using the two end-member mixing model^39^:

1 $${{\rm{f}}}_{{\rm{C}}3}=\frac{{{\rm{\delta }}}^{13}{{\rm{C}}}_{{\rm{SOC}}}-{{\rm{\delta }}}^{13}{{\rm{C}}}_{{\rm{C}}4}}{{{\rm{\delta }}}^{13}{{\rm{C}}}_{{\rm{C}}3}-{{\rm{\delta }}}^{13}{{\rm{C}}}_{{\rm{C}}4}}\,$$

2 $${{\rm{f}}}_{{\rm{C}}4}=1-{{\rm{f}}}_{{\rm{C}}3}\,$$

where δ^13^C_SOC_ represents the stable ^13^C composition in SOC, δ^13^C_C3_ in wheat and δ^13^C_C4_ in maize. According to Boutton^40^, average δ^13^C in wheat and maize are approximately −27‰ and −13‰, respectively.

We estimate that the contributions of wheat and maize to SOC are 61.3- 68.1%, and 31.9–38.8%, respectively (Table 3). There are no significant differences in the relative contributions of maize and wheat to SOC between layers in soil profiles far away from the Yellow River, but significant (P < 0.05) differences between topsoil and subsoil near the river. Our data suggest that the contribution of wheat is significantly (P < 0.05) larger in the subsoils near the Yellow River.

**Table 3:** Mean values (standard deviations) of contributions of maize and wheat to SOC in the whole study area, near the Yellow River and far away from the Yellow River at different soil layer. *Values followed by the same letter (low case letter within a column or upper case letter between near the Yellow River and far away from the Yellow River) are not significantly different at P < 0.05 based on LSD test.

Depth (cm)	Maize-SOC (%)			Wheat-SOC (%)		
	Whole area	Close	Far away	Whole area	Close	Far away
0–20	37.6 (5.41) a	35.7 (3.61) Aab	39.2 (6.35) Aa	62.4 (5.41) b	64.3 (3.61) Abc	60.8 (6.35) Aa
20–40	38.8 (6.80) a	37.7 (6.07) Aa	39.7 (7.56) Aa	61.3 (6.80) b	62.3 (6.07) Ac	60.3 (7.56) Aa
40–60	38.4 (7.14) a	37.5 (5.00) Aa	39.2 (8.82) Aa	61.6 (7.14) b	62.5 (5.00) Ac	60.8 (8.82) Aa
60–80	36.0 (8.86) ab	31.3 (5.05) Bb	40.3 (9.57) Aa	64.0 (8.86) ab	68.7 (5.05) Ab	59.7 (9.57) Ba
80–100	31.9 (9.30) b	26.4 (5.49) Bc	37.0 (9.35) Aa	68.1 (9.30) a	73.6 (5.49) Aa	63.1 (9.35) Ba

Wang *et al*. (2015) reported that the contribution of wheat to SOC is 60–72% in other wheat-maize double cropping systems of North China^27^. A long-term experiment in wheat-maize cropland of Mexico (with similar climate) also indicated greater contribution to SOC by wheat residues^22^. Earlier studies suggest that maize residue has a rapid decomposition rate, probably resulting from the low lignin content and low C:N ratio and relatively higher temperature during the growing season^27,41^.

### Influence of the hydrological processes

Our analyses indicate that the Yellow River has significantly influences on SOC, TN, and δ^13^C in subsoil. In particular, mean content of SOC below 80 cm and TN below 60 cm are significantly lower near the river than far away (P < 0.05, Fig. 2). Here, we explore possible mechanisms that may be responsible for the lower levels of SOC and TN in the subsoil near the Yellow River, which may include biogeochemical processes (e.g., enhanced decomposition of SOM) and/or hydrological processes (e.g., enhancement in dissolution and transportation due to water movement).

We first assess the possibility of enhancement in SOM decomposition. The clay fraction is relatively higher near the river, which makes decomposition of SOM difficult due to protection by aggregation^10,42^; salt content is comparatively higher in the soil near the river (Table 1), which would inhibit microbial activity thus decomposition of SOM^30,31^. These analyses rule out the possibility of enhanced decomposition of SOM near the river. In addition, the more negative δ^13^C value in SOC of subsoil near the Yellow River confirms the impossibility of enhanced decomposition (which would lead to enrichment of ^13^C in SOC^26^).

We then evaluate the possibility of the hydrological impacts. There is evidence that precipitation near the river (651 to 750 mm) is more abundant than far from the river (551 to 650 mm)^43^, implying stronger hydrological processes near the river. Apparently, the hydrological influence on the subsoils would be more intensive than the topsoils, due to the linkage to the underground water. Based on the above analyses, we postulate that the hydrological processes (e.g., dissolution and transportation of SOM) may be partly responsible for the lower SOC and TN contents observed in the subsoils near the river. While there is no direct measurement of relevant variables, there is evidence of significant amounts of organic carbon in the Yellow River^32^, which could indirectly support our inference.

Precipitation occurs mainly during the period of July-August in the North China Plain^44^, and the river runoff is profound in summer^45^. The associated hydrological processes during the summer maize’s growing season would lead to more maize residues removed thus relatively more wheat residues remaining in soil profiles. The more negative δ^13^C in the subsoil near the river confirms that there is more wheat derived SOC. Such hydrological impacts would exist widely in the North China Plain, but be more pronounced near the Yellow River, which is supported by our data and an earlier study^27^ that demonstrate more negative δ^13^C values in the soil profiles near the Yellow River. More studies that involve hydrological processes are needed to better understanding the underlying mechanism regulating the carbon cycle in the cropland of North China Plain.

## Conclusions

We have investigated spatial large-scale distributions of SOC, TN, soil C:N ratio and δ^13^C in SOC over 0–100 cm in the representative wheat-maize cropland of the North China Plain. Compared with other croplands in China, SOC is relatively lower, but TN comparable in the North China Plain. As a result, soil C:N ratio is relatively small (9.4) in the North China Plain. The contribution of wheat to SOC (61.3–68.1%) is almost double of the maize’s contribution (31.9–38.8%).

There are significant differences in the subsoils’ characteristics between the near river and far away groups. SOC and TN contents are lower, and δ^13^C value is more negative near the river than far away. Our analyses imply that the low levels of SOC in the North China Plain may be partially owing to the relatively low soil C:N ratio, and the low SOC in the subsoils near the Yellow River could be linked to the hydrological processes (e.g., through underground water movement and river runoff).

## Materials and Methods

### Characteristics of the study region

The studying area, the main part of the North China Plain, is characterized by a typical continental temperate monsoon climate, with a mean annual air temperature ranging from 12 to 14 °C and precipitation from 550 to 750 mm. The rainfall, largely concentrated in the season of June-September, increased from northwest to southeast^43^. The elevation in the study area shows little variation, ranging from 63 m to 4 m. The studying area covers almost 80 thousand square kilometer, but the soils are all developed on alluvial loess. The soils are Ochri-Aquic Camosols and Endorusti—Ustic Cambosols according to the Chinese soil classification system (1995), which are classified as the Calcaric Cambisol and Fluvo-aquic in the FAO-UNESCO system (1988)^9^. The groundwater table is gradually increasing, and salinity in the shallow groundwater and soil is increasing from the west plain to east coastal plain^33^. The main cropping system is a double rotation of winter wheat (Triticum aestivum L.) and summer maize (Zea mays L.). Based on our interviews with farmers, agricultural management (tillage, irrigation and fertilization and so on) are very similar in the study area. Farmers often use mixed mineral nitrogen-phosphorus-potassium fertilizers, and irrigate with underground water in the area far away from the Yellow River and with groundwater and river water near the Yellow River.

### Soil sampling and analyses

In our previous works, we sampled at 31 sites in the upper Yellow River Delta in August 2015^28^ and 24 sites in Hebei Plain in early October 2015^9^. In this study, we chose representative sites: 10 sites near the Yellow River and 21 sites far away. At each site, 3-4 soil profiles were randomly selected. We collected soils over 0–20, 20–40, 40–60, 60–80, and 80–100 cm (using 5-cm diameter soil augers). Soil samples were air–dried, well mixed and sieved to pass a 2-mm screen. The determinations of pH, EC, TDS, soluble Ca^2+^ and Mg^2+^, SOC and TN were reported by Shi *et al*. (2017)^9^. Stable ^13^C isotope was determined by measuring the isotopic composition of collected CO_2_ using a Finnigan MAT Delta Plus XP Isotope Ratio Mass Spectrometer. The analyses of total soil nitrogen, SOC and stable δ^13^C isotope were performed at the State Key Laboratory of Lake Science and Environment, Nanjing Institute of Geography and Limnology, Chinese Academy of Sciences. We reported isotope data in delta notation relative to the Vienna Pee Dee Belemnite (VPDB).

### Statistical analyses

We used Fisher’s protected least significant difference (LSD) to compare all parameters between different soil layers, including SOC, TN, soil C:N ratio, δ^13^C, wheat-SOC and maize-SOC. All analyses and figures were performed using SPSS (version 22), Sigmaplot (version 12.5) and Arcgis (version 10.1).
